# Keeping up with the caravan of life: Successful aging strategies for Iranian women

**DOI:** 10.3402/qhw.v10.29500

**Published:** 2015-11-24

**Authors:** Nazila Javadi-Pashaki, Farahnaz Mohammadi, Fateme Jafaraghaee, Neda Mehrdad

**Affiliations:** 1 Social Determinants of Health (SDH) Research Center, School of Nursing and Midwifery, Guilan University of Medical Sciences (GUMS), Rasht, Iran; 2 Department of Gerontology & Nursing, University of Social Welfare and Rehabilitation Sciences (USWR), Tehran, Iran; 3 Elderly Health Research Center, Endocrinology and Metabolism Population Sciences Institute, Tehran University of Medical Sciences, Tehran, Iran; 4 Endocrinology and Metabolism Research Center, Endocrinology and Metabolism Clinical Sciences Institute, Tehran University of Medical Sciences, Tehran, Iran

**Keywords:** Iran, grounded theory, successful aging, women

## Abstract

**Background:**

Because of improving life expectancy in the world in recent times, the focus has shifted to the issue of the quality and nature of life and how to assist successful aging (SA) rather than increasing physical survival and lifespan. SA is a multidimensional, relative, and context-dependent concept with different paths and outcomes. The purpose of this qualitative study was to explore older women's strategies for SA in a specific context.

**Methods:**

Following a grounded theory design approach, we conducted semi-structured individual interviews with 21 women between the ages of 28 and 96 years. We analyzed the data from interviews, written narratives, and field notes using the grounded theory approach.

**Results:**

We identified four categories: *prevention of threats, internal self-control against threats, coping with threats, and optimizing the passage of time according to opportunity*. These described the strategies for SA when encountering with age-related changes. Utilizing these strategies, the women accompanied the caravan of life in the context of threats and opportunities.

**Conclusions:**

The findings suggest that SA is a continuous process in confronting changes related to age. The identified strategies can help to promote SA by familiarizing older women with the threats and opportunities of life and training them in how to use these strategies.

Currently, as a result of improvements in life expectancy, the quality and nature of survival are considered an important issue around the world. Successful aging (SA) is an indisputable right for everyone in addition to increased longevity. Understanding of SA and exploration of its strategies are necessary for caring for elderly people and creating supportive as well as preventive interventions (World Health Organization, 2015). SA is a multidimensional and relative concept that depends on the cultural context, values, and norms related to the culture and society in which each individual lives (Wakasaki, Mutsomoto, & Kakehashi, 2006; Willcox, Willcox, Sokolovsky, & Sakihara, 2007). Considering the different meanings of success in each community, SA may follow different paths leading to different outcomes. Therefore, theories and models formed in a particular community may not be applicable for other communities. With its aging population, Iran is a country with a specific cultural context different from other countries. Health culture in Iran is more based on treatment rather than prevention. The majority of elderly women in our society do not have financial independence. Therefore, they have a certain lifestyle that can affect the way they age (Samarakoon, Chandola, & Ravishankar, 2011). So far, no studies have been conducted to identify strategies for successful aging in our country.

SA is a fairly subjective concept, as the understanding of success depends on the presuppositions and inner experiences of individuals. Therefore, no quantitative approach can definitively determine the reality and circumstances of SA. We have thus undertaken as deep and comprehensive a study of this phenomenon as possible via qualitative approaches. Among other qualitative research designs, grounded theory investigates social processes in the field of reality and is the most appropriate method for study processes and experiences appeared in various forms (Munhall, 2007). On one hand, due to the longer life expectancy of Iranian women (71.7 years for males vs 76.5 years for females), they currently make up a larger portion of the elderly population in Iran (Statistical Center of Iran, 2011). On the other hand, women may have very different life experiences because of the differences between their roles and those of men in Iran. Women experience illness, stress, and disability more than men. However, they have greater longevity. Studies suggest that the personality characteristics, unique perspectives, and lifestyles of women may contribute to their health status, high flexibility, and successful aging (Javadi Pashaki, Mohammadi, Jafaraghaee, & Mehrdad, 2015; Sheffler, 2011). Women can be considered good subjects to understand strategies for SA. This article is a part of a larger study where a grounded theory–based approach was followed to theoretically explain SA within Iranian women. In this paper, we report on one of the main themes, *accompanying the caravan of life in the shadow of threats and opportunities*, that showed women's strategies for SA.

## Methods

This qualitative study was conducted in Iran, from April 2013 to December 2014. The main participants were community-dwelling women over age 60 that were able to speak Farsi. Studies show that the personality characteristics, attitudes, and unique lifestyles of women improve their health and enhance their flexibility in old age (Hsu & Jones, 2012). Therefore, they can be a good example for understanding SA. Initially, according to the purposive sampling procedure, elderly women, who had been through the experience of aging, were invited to participate. These participants were selected purposively from three aging groups (young-old, old, and old-old). Based on a preliminary data analysis of participants during the theoretical sampling process, other women were included. After purposive sampling, we collected data from people who could provide the appropriate and relevant data for completion concepts, categories, and its relationships. Theoretical sampling was used to determine who to sample next and what questions to ask during interviews. After initial simultaneous data collection and analysis, we also found that SA strategies are not limited to the stage of aging. Thus women in other age groups were included. By theoretical sampling we selected non-elderly female family members to cover a broader range of behaviors. Based on the principles of maximum variation in sampling, participants came from a heterogeneous group of elderly people with differing perspectives and experience. All elderly participants functioned well cognitively, with a mean score of 7 or higher on the abbreviated mental test (Hodkinson, 1972). Elderly women were recruited from different settings, including Iranian retirement homes, physicians’ offices, city parks, elderly people's workplaces, and health centers affiliated with municipalities. Some women were approached by introducing previous participants. In all, there were 21 participants, aged 28–96 years, mean 52.67 years, who volunteered and consented to participate in the study (Table I).

**Table I:** Characteristics of the participants.

Participant number	Age	Education	Marital status	Number of children	Type of residence	Living with whom?	Employment status
1	61	Diploma	Married	5	Urban	Living with spouse	Housewife
2	70	Literate (reading and writing)	Married	5	Urban	Living with spouse	Housewife
3	86	Diploma	Widowed	7	Urban	Living with son and his family	Retired
4	65	Diploma	Widowed	3	Urban	Living with unmarried child	Retired
5	76	Elementary school (second grade)	Widowed	5	Urban	Living independently	Housewife
6	84	Diploma	Divorced	4	Urban	Living with unmarried child	Retired
7	63	Diploma	Married	—	Urban	Living with spouse	Retired
8	83	Illiterate	Divorced	5	Urban	Living independently	Housewife
9	70	Bachelor's degree	Married	3	Urban	Living with spouse	Retired
10	96	Doctoral degree	Widowed	3	Urban	Living with caregiver	Retired
11	66	Bachelor's degree	Single	—	Urban	Living independently	Retired (Returning to work)
12	81	Illiterate	Widowed	6	Rural	Living independently	Housewife
13	70	Literate (reading and writing)	Widowed	6	Urban	Living with spouse and unmarried child	Housewife
14	68	Diploma	Married	2	Urban	Living with spouse and unmarried children	Housewife
15	77	Literate (reading and writing)	Married	3	Urban	Living with spouse	Housewife
16	76	Literate (reading and writing)	Widowed	4	Urban	Living independently	Housewife
17	78	Illiterate	Widowed	3	Urban	Living in nursing home	Housewife
18	49	Diploma	Married	1	Urban	Living with an older relative	Retired
19	32	Middle school (third degree)	Married	1	Urban	Living with an older relative	Housewife
20	28	Bachelor's degree	Single	—	Urban	Living with an older relative	Employed
21	78	Illiterate	Widowed	3	Urban	Living in nursing home	Housewife

We conducted semi-structured interviews with open-ended questions based on an interview guide. A total of 21 interviews were conducted. Interviews lasted an average of 85 min in length (range 30–120 min). We audiotaped interviews and subsequently transcribed them verbatim. During each interview, we started with general and primary questions including “what comes to mind when you hear SA?” According to the responses, the interview guide was amended for clarification to the questions. “What do you do to age well?” During the interview, we asked additional questions, when needed, to elicit further details and to probe for more information while exploring the ideas expressed by the participants. Furthermore, the type and form of the questions went through a process of development to ensure that the topic and focus of the interview were developed alongside the analytical process. We arranged the interviews according to participants’ preferences. The interviews were conducted either at participants’ workplace or home. The interview ended when participants became fatigued.

Collecting of data was continued until relationships between the groups were clear and theoretical saturation was achieved. After coding and analysis for the final two interviews, all data were replicated until no new categories or concepts were added. In addition, the properties and dimensions of categories were determined and relationships between categories formed. Therefore, we stopped data collection due to theoretical saturation.

Data were analyzed through constant comparative analysis in which data were reviewed iteratively to identify new and emerging themes (Corbin & Strauss, 2008). We chose grounded theory as it is especially useful for uncovering processes and understanding participants’ lives and worlds. It is useful for the understanding of fundamental social–psychological patterns such as SA (Chenitz & Swanson, 1986). We conducted data collection and analysis simultaneously. Written field notes and memos were included in the analysis process to capture methodological and theoretical perspectives as they developed and to record ideas and assumptions regarding data and the relation and exposition between the categories. Initially the analysis process was a line-by-line reading of transcripts and identification of significant words or phrases (open coding that was at the most concrete level) in order to break the data into pieces for the purpose of precise analysis. Then, we met together for generating a general code list. During open coding we did a continuous comparison for similarities and differences in different parts of the data. We conducted focus and axial coding and discussed emerging themes, concepts, and new codes during the meeting. Focused coding that was designed to identify and clarify larger more prominent concepts followed this process. We used axial coding to connect the concepts that emerged and to form a theory. We grouped the codes into overarching themes, subthemes, and SA strategies. We also considered the properties and dimensions of each category as well as connections between categories. We continually compared all illuminated categories to each other until saturated by theoretical sampling; that is, the emerging results directed us to where new data could be found and which questions should be asked to add further information. Through the process of constant comparison (Corbin & Strauss, 2008) we further condensed the independent categories into discrete themes. We identified a core category, which was the most abstract level, and all other categories were related to it by means of a paradigm model that explored context, action, and interaction conditions, as well as the consequences involved in the process of SA. Finally, we wrote a story line describing the process.

We ensured truthfulness of the study through adherence to methodology, constant comparison of findings to participants’ expressions, and confirmation of the finalized theory with many participants. Prolonged engagement with the study and the data, maximum variation sampling, and member checking established credibility of the findings. Member and peer checking techniques were employed for enhancing the dependability and the confirmability of the findings. Accordingly, participants and two external qualitative researchers were asked to check the congruence between participants’ experiences and the findings. Finally, we strived to clearly describe the study sample and setting, to enhance the transferability of the study findings. In the spirit of the grounded theory approach, we had reflexivity in guiding the progress of the study, which added to the rigor of the approach (Corbin & Strauss, 2008).

The study was approved by the ethical committee of Tehran University of Medical Sciences (no. 3550). We briefed participants beforehand that the interview would consist of a discussion of their views on SA. Participants provided a written informed consent for the study including audiotaping and transcription of the interview prior to participation. We ensured their confidentiality in this study (Peter, 2015).

## Results

Four categories emerged from the data: (1) prevention of threats; (2) coping with threats; (3) internal self-control against threats; and (4) optimizing the passage of time according to opportunity (Table II). These described the strategies for SA when encountering age-related changes. *Accompanying the caravan of life in the shadow of threats and opportunities* was identified as the core category.

**Table II:** Categories and sub-categories in the study.

Category	Sub-category
Prevention of threats	Preventative self-care
	Positive interaction with others
	Proactive economic management
	Seeking independence
Coping with threats	Stopping behaviors that cause threats
	Modification of previous behavior
	Adjusting the environment to suit the change
	Compensating for losses
	Selecting alternatives
	Hardiness against change
Internal self-control	Seeing the bright side of life
against threats	Avoiding the dark facets of life
	Justification of facts
	Distraction from threats
	Staying hopeful
	Recourse to spirituality
	Fortitude against changes
Optimize passage of	Sweetening life
time according to	Using life opportunities
opportunity	Spiritual journey
	Optimal use of existing individual capacities

### Prevention of threats

The women encountered changes in the physical, cognitive, mood, role, social, and economic dimensions during the process of aging. They experienced changes personally or observed them in elderly people. The changes were the context for adopting SA strategies. The younger women prevented threats before they occurred. This strategy was used less frequently by the elderly, since they were threatened by changes anymore. They prevented from possible future threats. Anyway, they adopted preventative self-care, positive interaction with others, proactive economic management, and independence seeking.

#### Preventative self-care

The women became concerned with their physical, cognitive, and spiritual health after they perceived the bitterness and sweetness of their lifetime passing. By adopting a healthy lifestyle, searching through books and media for health information, as well as monitoring their health and getting periodic checkups, the women were caring for their health. Studying and learning activities to keep the mind active was a solution to prevent cognitive threats. In this way, they were safeguarded against possible physical, cognitive, and psychological threats they were prone to.

I take my medication. I have blood tests periodically. I'm not eating fatty and salty foods. I exercise to stop other problems from coming for me. I swim to prevent my joints from being dried. (70 years old)

#### Positive interaction with others

The women were trying to have consistent and positive relationships with their families and communities via providing restraint, patience, dignity, and well-adapted communication and a positive mood when dealing with others. In this way, they were avoiding loneliness, social isolation, and loss of the valuable roles these factors played in preventing subsequent mental disorders. Positive interaction and compatibility with others had led them to maintain their family and social networks while developing a communication network with their peers and younger generations. This role can be played in terms of providing advice to others, especially younger generations. Adaptation and positive interactions provided the context for getting dignity and respect from family and society.

#### Proactive economic management

The participants were prevented from possible future economic threats by their financial savings. Their savings and accumulated capital had helped them not to be dependent on their families, not to be forced to reside in nursing homes, and not to be rejected by their families in the event of disability by employing a caregiver. Economic problems would threaten the role they were playing, while their savings acted to protect them from such threats.

#### Independence seeking

Seeking independence and relying on their inner strength helped women remain immune from mental disorders due to dependence. Those who were lonely, widowed, and separated from their children had attempted to rely on themselves to maintain their internal integrity, so that their independence was maintained. Self-reliance actively contributed to threat prevention. Choosing to live independently maintained their actual independence and prevented potential conflict with family members. This helped them to preserve their dignity in the family and society.

### Coping with threats

When threats occurred, the women tried to actively control the change, situation, or behavior. Stopping the behaviors causing the threat, modifying previous behaviors, adjusting their environment to suit the change, compensating for the losses caused by the passage of time, and exhibiting hardiness against changes were all behavioral strategies adopted by the women to cope with threats so that they were keeping up with the caravan of life. The women used one or more strategies when faced with the threats of aging.

#### Stopping the behaviors causing a threat

When faced with changes, the women stopped the acts causing the threats or attempted to avoid exposure to toxic threats. This approach could be temporary or permanent. Avoidance or cessation of harmful activities or practices for health allowed them to avoid threats. In this way, it helped the women maintain their physical and mental health:

I went to their house to take care of my grandchildren while their parents went to work. But now I can't afford to continue it due to knee problems and orthopedic difficulties. I dropped it for my health and it was necessary. (65 years old)

#### Modifying previous behaviors

Correction and modification of behaviors based on changes experienced by the women were used to cope with the threat of aging. Contentment, sparing actions, and adjusting their activities before occurrence of a threat were behaviors for coping with life threats:

I don't pray on my knees; rather I prefer to say prayers while I'm seated. I use the toilet. I won't try standing long in the kitchen. (70 years old)

#### Adjusting the environment to suit the change

Modification of the state on the basis of lost abilities was a solution to cope with the threats. Based on changes in the aging experience, they regulated their living environment by inducing changes in the environment, placing personal items within their access, moving to a place of residence fitted to their physical abilities, and using facilities to fit the changing experience of their lives:

I can't do this hike. The doctor told me to be careful not to break my leg. I can't lay on my legs. I'm just sitting on the bed. My phone is here. There are things around me. The radio is at my side to stand less. (76 years old)

#### Compensating for losses

The women corrected losses or defects faced over the passing of time by using aids such as crutches, eyeglasses, and hearing aids. They were trying to compensate for losses and lacks caused by the passage of time with the help of others. Income problems were offset by using previous savings and secondary sources of income. Compensation for and correction of defects allowed them to cope with life threats. Physical problems and disorders were compensated for and corrected by meeting a doctor and adhering to his or her prescribed medical treatment and care. Using aids such as wheelchairs assisted individuals to be active in the home and in society. Asking for help from people for their daily activities had helped them to keep track of their daily lives:

When I want to go out, I go with my wheelchair as I can't walk. (96 years old)

#### Selecting alternatives

Replacement was a solution used by the women when faced with a problem or threat. The women tried to find another person to fill the place of their family if their husbands had passed away and they were separated from their children:

I was afraid of being alone at night. Afterward, for the lonely night, I rent out the house. The tenant was like my child. She was helping me. (70 years old)

If the women were unable to perform a useful activity and interest, they replaced it with other activities in accordance with the change.

#### Hardiness against change

Persistence, insisting on completing tasks, and sustained and persistent efforts to achieve goals were indications of the women's hardiness toward advancing threats through time. Hardiness was not only an independent solution for keeping up with the old caravan, but had also contributed to the successful adaptation of other strategies:

Occasionally, it happens that I'm not in the mood to take a shower, for example, but I do. I take a shower anyway. This is the onset of depression at this age. But I try not to let it happen to me at all. (63 years old)

### Internal self-control against threats

Psychological self-control against changes and possible or existing threats was one of the strategies for the women to be accompanied with changes in lifetime passing. This strategy was employed along with other strategies or alone to accompany the passing of life, especially when other strategies were not effective. This strategy could be applied before occurrence of any threats due to age-related changes to prevent the threats, and it could be maintained after the threat for self-control and preventing related threats from being realized.

#### Seeing the bright side of life

Positive outlook and positive thinking were a strategy for internal self-control against the threats associated with aging. Looking at the positive aspects of life, rather than the negative ones, helped their minds engage with changes. Focusing on their current capabilities and capacities and viewing the positive aspects of themselves as well as their lives, while looking for the positive aspects of losses, were strategies for dealing with changes and maintaining internal control. This strategy helped these people have a useful life by playing effective roles, despite changes.

#### Avoiding the dark facets of life

Intellectual avoidance as an internal self-control strategy included not thinking about their current weakness, difficulties, discomforts, negative past, and even the future. Remembering and thinking about the difficulties and negative aspects of their lives would lead to negative psychological reactions that could threaten women's mental health. Avoiding negative memories had helped the women to keep a positive mood. Having no negative perspective on aging may enable psychological self-control against aging-related threats. In this way, the women not only preserved their previous life process but also maintained their mental health, providing an enjoyable and dynamic life for themselves. Intellectual avoidance was also a mechanism used by the younger women to release their minds in order to think about their goals in the life:

I don't like to think about the disabilities of old age. When I think deep down about the later years asking ‘How old am I?’, or ‘What form am I in?’ I would have not a good feeling. I'm trying not to engage mentally with the bad traits of aging. I keep my mind open to progress and achieve the goals that I have set in my mind. (28 years old)

#### Justification of facts

The women justified the changes caused by their passing lifetimes and aging in order to enhance their psychological self-control. This step had helped them to more easily pass through steps of lifetime changes. Justification of the realities of aging had led them to accept age-related changes and their coming deaths through spiritual and mental self-control:

When I could no longer fast, I was very upset, I was crying, but little by little, I became used to it. Since then, I thought to myself; when I was young, I could fast. I went to Mecca, in the heat, I did the fasting. Now it is natural that I can't manage it at this age. I came to accept that these are the natural paths of life. (66 years old)

#### Distraction from threats

The women would distract their minds from perceiving threats by engaging in various activities. Gardening, sewing, creating art, cooking, doing household chores, studying, making pilgrimages and journeys, going to parties, meet and greets, watching TV, and reading the Quran and saying prayers were some of the activities undertaken by the women for entertainment and distraction from the associated threats of age-related changes and also to provide psychological self-control against the threats of aging:

In order not to come to consecutive negative thoughts about myself, I busy myself somehow. I entertain myself with carpet weaving. It gets me rid of the uncomfortable thought that my life is going to be over (61 years old).

#### Staying hopeful

Hope was found to be helpful for mood control in older women faced with the threats of aging. They were hopeful about the progress of science and technological innovation, so as to improve the lives of seniors in the future. This reduced their individual concerns about potential threats in the future. Hope was a useful strategy to motivate them and give them some interest in life. With hope, individuals were directed toward keeping up with their passing lifetimes by controlling the threats that had led to their departure from the way they were living. Hope for a better future was not defeated by aging-related threats. Hope for the future had secured women from mental disorders and maintained their psychological health:

When I see old people in nursing homes, I say wow, is that where I will finally end at? I will be sad; however, I have my own set of hopes. I don't know what will happen tomorrow. Then, I shouldn't bother myself, or be depressed. I should be hopeful for tomorrow. Maybe in the future I will be 80, I'm 90 years old, and meanwhile, I won't be powerless. (66 years old)

#### Recourse to spirituality

Recourse to spirituality and reliance on a superior power (God) were mechanisms used by the women to deal with age-related threats throughout their lives and to achieve psychological self-control. Trust in God was triggering activity, despite the change. Women who trusted in God were able to overcome threats due to age-related changes and thus to keep up with the caravan of life. Recourse to the Quran enabled mental relaxation when faced with threats of aging. Due to an increased understanding of life, the desire for spirituality and religious activities was increased with rising age. Entrusting themselves to the honor of God and his superior power had contributed to their satisfaction and inner peace:

I entrust myself to God, being glad to do whatever is pleasing to God. It is comforting to me. (76 years old)

#### Fortitude against changes

Patience with the problems of age helped them to control themselves psychologically to deal with the threats of aging. In this way, they maintained their mental health. Patience was a passive approach toward the changes. Tolerance to physical changes helped individuals adapt to age-related changes. Patience helped them establish calm while providing compatible interactions with others:

I try to be patient. Being patient, you would be calm in your heart. Even a person who treats you badly, be calm. When you see he is quiet and you get style. (61 years old)

### Optimizing the passage of time according to opportunity

Along with psychological control and coping strategies, the women were trying to spend their lives in the best way through living opportunities. This strategy could be used if a person were not threatened or once she achieved self-control and coped with the threats. As long as threats and the bitterness of life were prevalent, individuals would engage deeply with them, as they would have no opportunity to optimize their passage of life. After realizing psychological self-control and coping with life threats, the women found the opportunity to optimize their lives by creating happiness, optimally utilizing their individual capacities and life opportunities, and also walking a spiritual path. Those women who were successful in coping with threats to life and self-control tried different methods to optimize their capabilities and opportunities in their lifespans when moving along with the caravan of life.

#### Sweetening life

One of the opportunities provided by aging was to create teasers illustrating the sweetness of life. Variety in their lives gave the women vitality. Participation in happy, friendly and family communities, reviewing happy memories, within intimate communities, provided the women with joy. Using this strategy, women did not feel changes in the current trend compared to before. Creating an enjoyable situation brought the women satisfaction and psychological health:

I'm looking for something that makes me happy at this age. I'm travelling, listening to music, going to restaurants with my friends, so that I will be so charged. I will be happy. I'm very happy to meet some old friends. I wear clothes that I love. I should do all of these and repeat so that I do not lose myself in aging. (63 years old)

#### Using life opportunities

When faced with changes over time, some participants saw aging as an opportunity that could be used optimally, once they succeeded in taking psychological control and coping with threats. Optimal use of the leisure time provided by the passing of life is to pursue interests and activities such as sports and art classes, pilgrimages and tourism trips, and friendly meetings:

I was busy looking after my husband until he died. Now that I have more free time, I can go anywhere. I even decided to go to classes. These days I consider myself a bit more. (65 years old)

#### Spiritual journey

Some women had chosen to follow a spiritual path based on spiritual desires, goals, and practices to optimize their aging. Spiritual journeys could occur at any period of life, including older age. Sometimes exposure to aging and aging changes triggered individuals to take steps in this direction. Based on their spiritual interests, the women chose to optimize their spiritual path by taking certain actions and practicing spiritual activities. These actions included helping poor people, responding to help requests, and supporting charities. Aging made them feel that they must pay attention to their afterlife, so that they insistently went for religious practices:

I have a life full of content. I would help everyone, as far as I can. I don't want to let anyone down. Their prayers are enough for me. I have already done some charity work. Material issues are not important to me. Not already. I am comfortable with the end of life. (86 years old)

#### Optimal use of existing individual capacities

Use of existing capacity was an optimization approach to life in the shadow of chance. Experiences and skills gained during life helped individuals optimize their remaining lifetime, despite losses. The use of existing capacities included performing useful activities and getting as much benefit from family as possible, creativity, teaching and learning activities, sharing experiences with others while trying to learn a new skill (e.g. art). It was possible to use this potential in the shadow of a chance. Teaching and learning activities and creating works of art had a positive effect on the continuation of the current activities and routines of life and psychological state of the individual. Through activities such as those mentioned, women used their mental and physical capacities in order to perform their roles effectively:

Now, I sit here and prepare clean vegetables for my daughter. I call her to come and take them. So what I can, I do. I don't want to spend my time in vain. I want to do everything in my power. (76 years old)

## Discussion

We explored how the women adopted strategies to cope with threats and opportunities to keep up with the caravan of life during the SA process. The women, especially the non-elderly, prevented threats by seeking independent behaviors, preventive economic management, preventive self-care behaviors, and positive interactions with others. In Iranian health culture in the past, prevention was not an important issue. Thus, elderly women rarely used prevention strategies before aging. However, based on new health policies, prevention is now much more important among young women (Ebrahimi Tavani, Ghofranipour, Hajizadeh, & Abedini, 2015).

Independence is a cognitive behavioral mechanism to avoid dependence on others and maintain the dignity of the elderly, which helps them maintain their inner integrity (Ryff, 1989). According to Kahana and Kahana (2001), preventive strategies are used prior to the occurrence of stressors in order to overcome them or reduce their number so their negative consequences can be avoided. These precautionary strategies include health promotion to reduce the risk of health problems, planning for the future, and helping others in order to further enrich social resources. Once a problem occurs, health promotion and preventive activities to proactively cope with the problem improve quality of life for the elderly (Ouwehand, De Ridder, & Bensing, 2007). The primary control strategy is the proactive effort to provide the resources to achieve an individual's important goals in life (Baltes & Baltes, 1990). In line with our findings, in a study by Lachman (2006) the elderly also used preventive control strategies as control mechanisms. Health promotion activities to maintain good physical health and functional mobility are examples of mechanisms that have been introduced as an SA process (Blevins & Troutman, 2011).

In our study, involvement with age-related changes and their psychological consequences served as the context for using internal self-control mechanisms via seeing the bright side of life, avoiding the dark spots of life, staying hopeful, disregarding bad memories, justifying the facts, using spirituality, and being patient. Internal self-control was a kind of psychological control when faced with changes. Personal control is an inner psychological coping factor (Blevins & Troutman, 2011). The results of our study are in accordance with those of other studies showing that the following strategies are being used by the elderly: independence (Dunéra & Nordstromb, 2005; Huang & Acton, 2009), patience (Lavretsky & Irwin, 2007), positive thinking (Blevins & Troutman, 2011), hope (Zhang, 2012), avoiding subjectivity (Bagheri-Nesami, Rafii, Seyede Fatemeh, & Oskouie, 2010), spiritual orientation (Vance, Struzick, & Raper, 2008), and spending leisure time (Bagheri-Nesami et al., 2010). In fact, these strategies are coping strategies that contribute to a good life at all stages of life.

Once a change occurred, our participants either corrected and compensated for the change or its situation, or adjusted and stopped the behavior. They were resistant in their efforts to cope with threats. In one study elderly individuals used compensational control strategies and behavioral therapy as control strategies against changes (Lachman, 2006). In our study the women replaced unfavorable conditions with appropriate conditions and actions. Activity theory suggests that a component of SA is replacement of the age-related losses in order to maintain a positive sense of self (Havighurst, 1963). Compensation, replacement of achievable goals, and keeping away from experiences of failure constitute the mechanism of the compensatory secondary control strategy. People compensate for a loss in order to adapt to changes in the biological, psychological, and socioeconomic evolution throughout their lives and to create a suitable environment for lifelong success (Baltes & Baltes, 1990). According to Flood's theory of successful aging, if there is a tool related to a selected target, an elderly individual will pick it up. If not, instead of choosing tools for other purposes, the individual will use replacement instruments to achieve a specific goal (Blevins & Troutman, 2011). Our participants gave up undesirable actions while avoiding exposure to threatening situations in order to cope with threats. The theory of disengagement suggests that SA is the willingness and ability of individual to retire from active life in order to prepare for death. Indeed, evaluation of blocked goals results in the resignation of the goals in the elderly (Ouwehand et al., 2007). Avoidance is an adaptive mechanism that might be used to relieve experienced distress and provide protection or conservation of resources. It is an attempt to either leave or stay away from stressful situations (Bagheri-Nesami et al., 2010). Tenacity and resistance against problems was another approach that participants followed to cope with threats. Tenacity and strength for achieving goals are a mechanism for achieving well-being, satisfaction, happiness, hope, optimism, and joy in the elderly. Strength and hardiness when facing a change are the opposite of flexibility and help individuals adjust to the changes of aging and achieve the intended objectives (Hill, 2011). Resistance is a self-management strategy to maintain one's function in terms of personal and social activities (Heo, Culp, Yamada, & Won, 2013; Katz, 2005).

Our participants optimized their passing lives by using their existing capacities and capabilities to make a joyful situation that would enhance the sweetness of their lives. This strategy is not clear in other studies and seems to be a notable finding in our study. Thus we suggest optimizing resources and facilitating success in selected and important areas in life. Indeed, attempting to maximize gains is an SA strategy for setting and following personal goals, providing adequate and appropriate resources, and also effectively using resources (Ouwehand et al., 2007). Creation of happiness was one of the solutions chosen by the elderly in our study to achieve gerotranscendence. The creation of joy in life contributes to psychological health. Seniors who sought to create happiness in their old age enjoyed good psychological health (Imperio, 2006). The women convoyed the caravan of life by using the opportunities of aging and moving toward or continuing on a spiritual path. Spiritual journeys were not always initiated by aging. However, they represent a move that sometimes occurs throughout life and is intensified in the elderly. Religious spirituality was the most important and valuable strategy for women's SA. Spirituality is one of the known processes for SA. It is used by women more than men; women have stronger religious tendencies (Zastrow, 2013). Spiritual activities are proactive adaptive behaviors performed by seniors. They reach toward self and life satisfaction by chasing transcendence in a spiritual direction (Imperio, 2006). Seniors with spiritual and religious tendencies have higher levels of autoregulation as well as health, happiness, and life satisfaction, leading to longer life spans (Udhayakumar & Ilango, 2012).

Family, especially children, had a strong role in all strategies. Despite the difficulties, inabilities, and lack of individual success in the elderly, older Iranian women are successful due to the success and happiness of their children. So for the elderly a child's success was their success. Single women were replacing other family members to child who have not. Thus, having a supportive family wherein children are very important is an essential component of SA strategies in Iranian women. This is justified by Iranian culture, in which women are heavily dependent on their children and family is a cohesion network.

## Conclusions

Women are subjected to changes with positive or negative consequences throughout their life. They implement appropriate strategies considering their sound understanding of the threats and life opportunities. In the process of SA, the volume of changes and threats are less important than the perception of changes related to aging. Perception of the aging situation is the basis on which one adopts strategies for SA. To the best of our knowledge, this matter is not seen in SA models known in the literature. Along with the caravan of life and beyond threats and opportunities is an ongoing process performed by women for being successful in their passing lives. It is based on their perceptions of changes related to aging. Accompanying the caravan of life is the motivational context within which to use preventive, coping, self-control, and life span optimizing strategies in order to have an SA experience.

Considering seniors’ capacities and life opportunities, introducing these opportunities and capacities to women and training them how to effectively implement their personal capacities and life opportunities could be an effective strategy for SA women. Policymakers in the field of elderly health should consider, design, and implement programs to empower older women to follow prevention strategies, exercise internal self-control, cope with threats, and optimize aging beyond threats and life opportunities. Teaching the strategies found in this study may help women in all age groups, and in particular older women, accompanying the caravan of life when faced by changes brought on by age, in the shadow of opportunities and threats to life, so that they can age successfully.

This study explored and described the SA strategies of a group of women within a specific context. The findings reflected their perspectives and experiences and cannot, therefore, be generalized to other populations. The human element in qualitative research is its weakness because it depends heavily on the researcher's skills, creativity, training, and intellect. Nevertheless the researchers did their best to control it.
